# Computational Nanoscopy of Tight Junctions at the Blood–Brain Barrier Interface

**DOI:** 10.3390/ijms20225583

**Published:** 2019-11-08

**Authors:** Nandhini Rajagopal, Flaviyan Jerome Irudayanathan, Shikha Nangia

**Affiliations:** Department of Biomedical and Chemical Engineering, Syracuse University, Syracuse, NY 13244, USA

**Keywords:** claudin, tight junctions, blood–brain barrier, in silico, drug discovery, membrane proteins, protein interactions, molecular dynamics

## Abstract

The selectivity of the blood–brain barrier (BBB) is primarily maintained by tight junctions (TJs), which act as gatekeepers of the paracellular space by blocking blood-borne toxins, drugs, and pathogens from entering the brain. The BBB presents a significant challenge in designing neurotherapeutics, so a comprehensive understanding of the TJ architecture can aid in the design of novel therapeutics. Unraveling the intricacies of TJs with conventional experimental techniques alone is challenging, but recently developed computational tools can provide a valuable molecular-level understanding of TJ architecture. We employed the computational methods toolkit to investigate claudin-5, a highly expressed TJ protein at the BBB interface. Our approach started with the prediction of claudin-5 structure, evaluation of stable dimer conformations and nanoscale assemblies, followed by the impact of lipid environments, and posttranslational modifications on these claudin-5 assemblies. These led to the study of TJ pores and barriers and finally understanding of ion and small molecule transport through the TJs. Some of these in silico, molecular-level findings, will need to be corroborated by future experiments. The resulting understanding can be advantageous towards the eventual goal of drug delivery across the BBB. This review provides key insights gleaned from a series of state-of-the-art nanoscale simulations (or computational nanoscopy studies) performed on the TJ architecture.

## 1. The Blood–Brain Barrier

**Figure 1:**
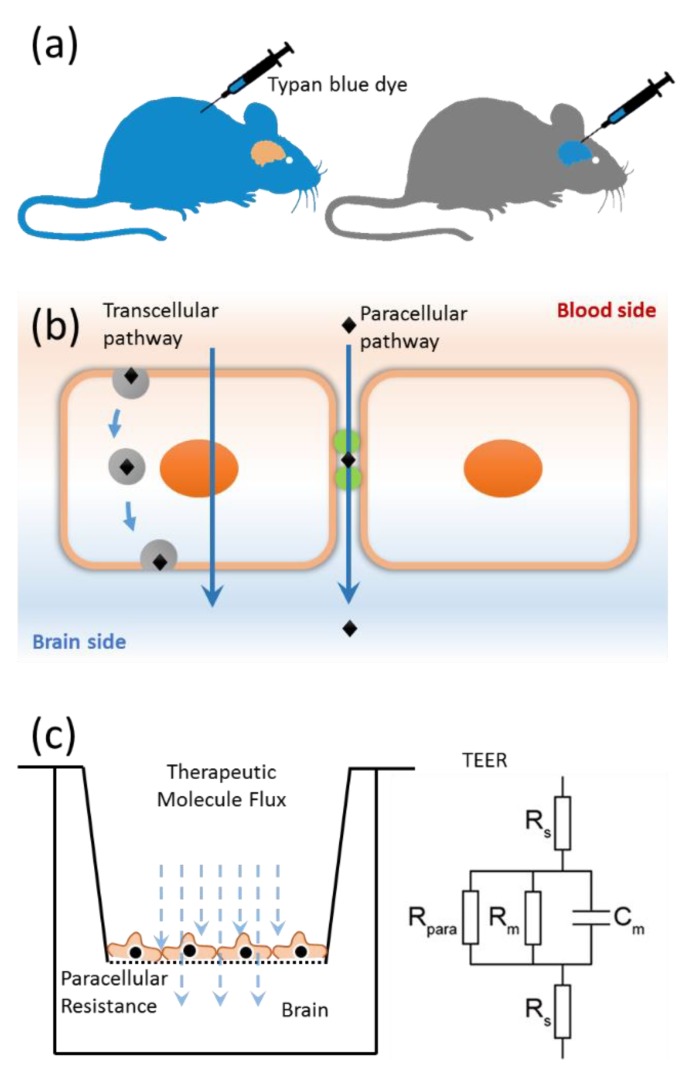
The blood–brain barrier. (**a**) Discovery of the blood–brain barrier (BBB), (**b**) pathways of molecular transport across the neurovascular endothelial barrier: transcellular (arrow through the cell) and paracellular (arrow through space between the two cells) and (**c**) in vitro characterization of the paracellular barrier strength of movement of therapeutic molecules (downward arrows) using transepithelial electric resistance (TEER) model.

The blood–brain barrier (BBB) is a selective permeability barrier that impedes the influx of potentially harmful blood-borne chemicals into the brain [1,2,3,4]. Although discovered by Ehrlich in 1885, the term blood–brain barrier was coined almost four decades later in the 1920s, after a series of reports by Lewandowsky, Goldmann, Stern, and Gautier [5,6,7,8,9,10,11,12,13,14,15,16,17,18,19,20,21,22]. Their collective work in vertebrate brain showed that tracers and toxins were unable to permeate the blood–brain interface; for example, trypan blue dye injected into the brain of a mouse remained confined to the brain, whereas the same dye injected outside the brain stained the entire body except the brain (Figure 1a). This permeability barrier is considered an evolutionary adaptation in vertebrates, and is vital for maintaining homeostasis in the CNS [1,5,21,22,23]. Unlike other organs, the brain lacks energy reserves [24,25,26]; therefore, for normal function, it requires a constant supply of nutrients from the bloodstream [27,28]. The brain’s microvasculature regulates the transfer of essential nutrients while protecting the brain against small chemical imbalances, which can lead to serious disease pathologies [29,30,31,32,33].

A critical component of the BBB is a specialized physical barrier comprised of tight junctions (TJs) present along the apical side of endothelial cells [1,23,34]. These cell-to-cell adhesion structures act as gatekeepers of the paracellular pathway (Figure 1b) by regulating the passive diffusion of molecules and ions into the brain [23,35,36]. The establishment of TJs at the BBB leads to a high electric resistance (>1000–3000 Ω cm^2^) across the interface [37,38]. The presence of TJs also leads to cell polarization whereby the apical and basolateral sides of the endothelia are isolated [2,35]. The polarized endothelia control transcellular permeability by specific expression of transporters and receptor proteins in the apical side of the membrane (Figure 1b). In the event of disease pathologies, TJs are compromised, leading to the loss of barrier selectivity in both the transcellular and paracellular pathways [33,35].

Although the BBB is vital for neurological functions, its selective permeability is a deterrent to drug delivery into the brain [31,39,40,41]. The BBB obstructs effective treatment of neurodegenerative diseases such as Alzheimer’s and Parkinson’s, because therapeutics are unable to cross the barrier [39]. To develop new therapeutics, in vivo animal models provide an experimental environment that mimics the intricacies of human physiology. However, high cost and lack of complete translation of disease pathologies from animal models to humans make in vivo studies less effective for drug development. In vitro models are therefore being used early in a drug’s development to investigate its permeability across the BBB [42,43,44]. Drug permeability is typically studied using cell cultures grown on permeable membranes in a transwell, where the transendothelial/epithelial electrical resistance (TEER) is used to measure the change in the electrical resistance across the cellular monolayer (Figure 1c) [37,45]. The TEER measurements evaluate the permeability and integrity of the endothelial cell layer [45,46], but the static cell culture cannot mimic the fluid characteristics of the BBB. More recently, microfluidic organ-on-chip in vitro models have been employed for the assessment of TJ barrier properties. Efforts are underway to develop BBB-on-a-chip that can recreate the human BBB using cells derived from the brain endothelial cells [47,48]. Additionally, in silico models of the BBB TJs have been developed to reliably compute the permeability of ions and small molecules across the paracellular barrier [49,50,51,52]. With advances in computer algorithms and hardware, strategies are being developed to design novel therapeutics that can cross the BBB. A concerted in vivo, in vitro, and in silico effort is underway to unravel the complexities of the BBB.

## 2. Tight Junctions: Role of Claudin-5 in the BBB

**Figure 2:**
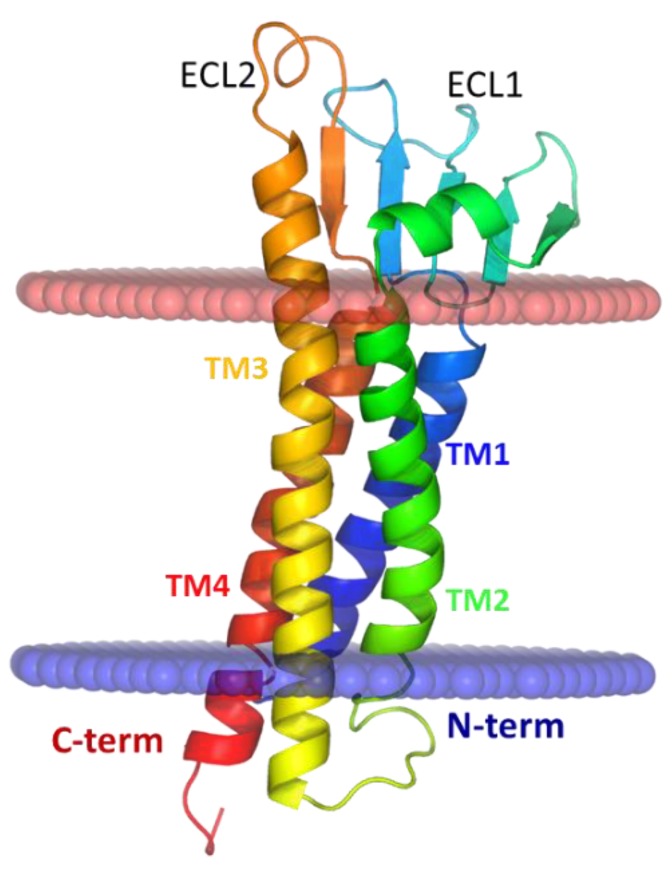
Homology modeled claudin-5 structure shows four transmembrane helices (TM1−4), extracellular loops (ECL1 and ECL2), and intracellular loop (ICL) in cartoon representation colored from N-terminal (blue) to truncated C-terminal (red). The membrane spanning region lies within the horizontal (red and blue) planes.

Claudin family of membrane proteins establishes TJs in endothelial and epithelial cells [53,54,55]. Claudins are expressed in vascular endothelial cells and all known epithelial cells throughout the body [56,57,58]. To date, 27 members of the claudin family are known to be functionally expressed in mammals [59,60]. They are classified into classic claudins (1–10, 14, 15, 17, and 19) and non-classic claudins (11–13, 16, 18, and 20–27) based on their sequence similarity and structural homology [59,61,62]. Most tissues express multiple members of the claudin family at the TJs [56,63,64,65,66,67]. Claudin proteins (~20–30 kDa) fold into a four-transmembrane helix bundle (TM1–4) with two extracellular loops (ECL1–2) and an intracellular loop (ICL; Figure 2) [60,62,68]. The lateral interactions mediated by TM and ECL domains of claudins within the membrane of the same cell are termed cis interactions. Cis interactions between multiple claudins occur in membranes of expressing cells. Subsequently, the claudins undergo trans assembly via head-on interactions of their ECL loops and form the macromolecular TJ assembly with adjacent claudin expressing cells. The TJ strands (often observable under freeze-fracture microscopy) are multi-component structures with claudins as the primary component alongside other TJ-associated proteins such as occludin, tricellulin, junctional adhesion molecules (JAMs), and zonula occludens (ZO-1,2) [53,60,61].

Claudin-5 is the key TJ protein at the BBB with an expression level that is much higher than claudin-1, -3, and -12 [69,70]. Knockout experiments have shown that claudin-5 is responsible for controlling the paracellular permeability of molecules up to ~800 Da [37,38,71]. In vitro biochemical analyses have suggested that claudin-5′s ECL2 loop strongly contributes to the trans interactions [72,73]. Mutations to conserved residues in ECL2 lead to a marked increase in paracellular permeability because of compromised size selectivity [59,74]. Studies also show that claudin-5 knockout mice die within 10 h of birth, although their TJs remain impermeable to molecules >800 Da in size [71]. In contrast a double knockdown study of both claudin-5 and occludin showed enhanced permeability of 3–10 kDa tracers indicating that occludin serves a structural role in the BBB TJs [75,76].

## 3. Need for Computational Modeling

In silico models complement in vitro and in vivo approaches used to investigate biological systems. Computer models augment our ability to analyze aspects of biological systems that are too complex to be investigated by conventional experiments alone [77,78,79,80]. Computational approaches provide a molecular-level basis for experimentally observed biomacromolecular assembly, selectivity, dynamics, and macroscopic properties.

For example, X-ray crystallography of a membrane protein provides essential structural information about its conformation at low temperatures [81,82]. However, proteins participate in dynamic processes and have thermal motions at physiological temperatures that cannot be resolved by current experimental techniques [83,84]. In silico methods can readily build upon the crystal structure data to provide information about molecular displacements, conformational dynamics, and reorientation frequencies of proteins [80]. The resulting models can also provide insights into the intramolecular and intermolecular interactions of a protein in its native environment, including other proteins, lipids, ions, and solvents. Another area where computational methods have been transformative is in predicting the three-dimensional structure of membrane proteins. The hydrophobic nature of membrane proteins makes them experimentally unwieldy for crystallization and X-ray structure determination [83,84,85]. In recent years, advances in protein sequencing have enabled the utilization of sequence co-evolution information to infer and predict the native fold(s) of a protein to a high-level of accuracy that can be validated by experimental biochemical assays [86,87,88,89,90].

Advances in computational hardware also have facilitated the study of complex biological systems. In the last decade, molecular simulations of biological systems with millions of interacting particles over microsecond to millisecond timescales have been reported [64,91,92,93,94,95,96,97,98,99,100,101,102,103,104,105,106,107,108,109,110,111,112,113,114,115,116]. These simulations provide unprecedented insights into the system under investigation and bridge nanoscale data to macroscale experimental results.

## 4. Computational Toolkit

**Figure 3:**
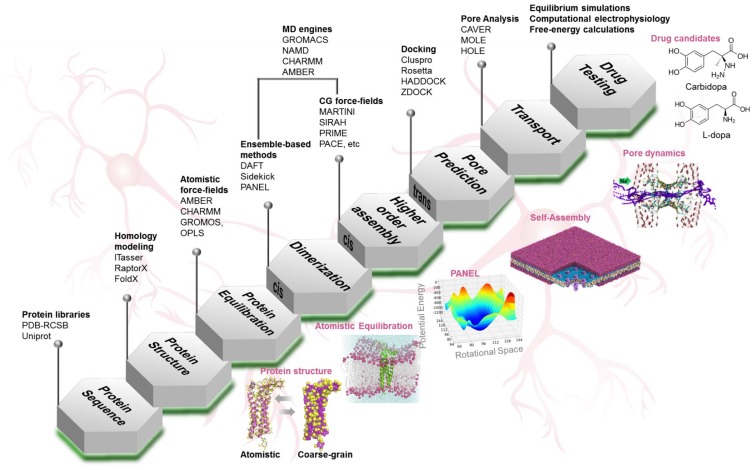
An overview of computational approaches and tools used to study the tight junctions at the blood–brain barrier. The stepwise research strategy included: obtaining protein sequence from online libraries; determining protein structure; equilibrating the protein in membrane lipids; investigating cis assembly (dimer and higher-order); investigating trans assembly for predicting tight junction barrier and pores; evaluating transport properties on ions and molecules; and testing tight junction modulators for enhanced drug delivery. The computational approaches (homology modeling, atomistic and coarse-grained molecular dynamics self-assembly simulations, PANEL, etc.) and tools (online libraries, webservers, force-fields, and software suites) associated with each step of our research strategy are also provided.

Computational methods, in the form of in-house computer programs, freely-distributed and commercial software suites, and online servers, offer an extensive toolkit that can be used to investigate different aspects of a biological system. Based on the desired qualitative or quantitative output, an array of computational models can be employed. Figure 3 summarizes a hierarchical computational approach relevant to the study of membrane proteins, using claudin-5 as an example.

### 4.1. Protein Structure Prediction

Crystal structures of murine and human claudins mClaudin-15 (4P79) [68], mClaudin-19 (3X29) [117], hClaudin-4 (5B2G) [118], and mClaudin-3 (6AKE) [119] are available. Most of the currently available claudin structures have missing loop and c-terminal domains, due to crystallographic artifacts. To study a claudin that has not yet been structurally resolved, the first step is to construct its homology model [120,121,122]. Reliable homology models can be derived if the target and template structures have at least 30% residue identity spanning the entire length of the domain of interest [120,123]. Since most claudins share ~30% sequence identity, this technique can reliably model structures of the other members of the claudin protein family [59,62,124]. Multiple tools are available online that can build homology models; [125,126] among them the I-TASSER [127,128,129] and RaptorX [130,131] servers are well established in the structural biology field (Figure 3). Both servers take the primary sequence of the target protein as input and give as output the lowest energy 3D-modeled structure of the protein. In building homology models care should be taken in modeling the nonconserved domains. For example, the flexible c-terminal domains of claudin family of proteins are challenging to model because they are intrinsically disordered and adopt nonspecific secondary structures [132]. Homology modeling methods are designed to provide a correct global structure but do not optimize side-chain orientations or flexible-loop conformations [121,132,133,134]. Such details can be obtained by loop modeling techniques such as ModLoop and FoldX [135,136]. Once the 3D-structure has been computationally determined, the next step is to embed the claudin in the desired lipid membrane and optimize the structure using molecular dynamics (MD) [137] simulation at atomistic resolution.

### 4.2. Protein Dynamics

Simulations of biological systems are performed at multiple resolutions—atomistic, coarse-grained, and mesoscale [137,138,139]. In atomistic resolution, the motion of each atom is evaluated as a function of time, whereas, in the coarse-grain (CG) resolution, a group of atoms in an amino acid is mapped to a bead to evaluate its dynamics. Mesoscale models take coarse graining up another notch; clusters of amino acids are mapped to a superbead, and simple harmonic oscillators represent the interaction between those superbeads [140,141,142]. Since each resolution has merits and pitfalls, multiscale modeling approaches are increasingly being used to take advantage of the benefits available at each resolution [143]. Multiscale simulations are vital to interpreting experimental results and guiding new lines of experimental investigation [80,144,145,146,147,148].

**Figure 4:**
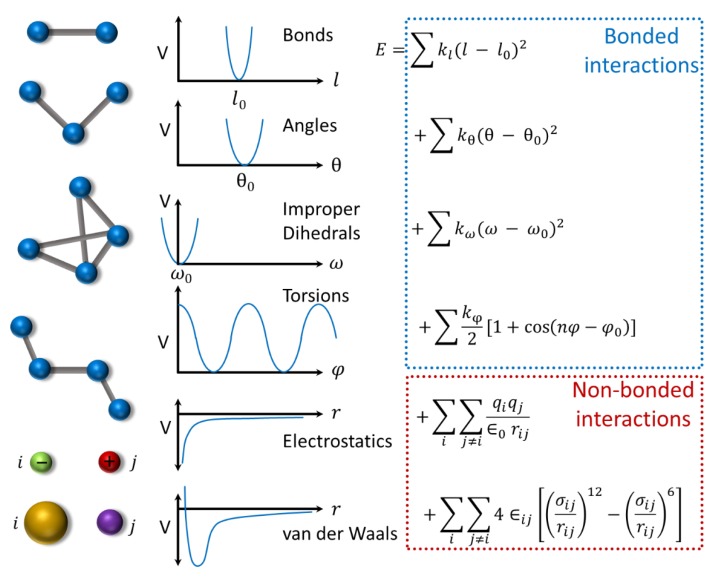
Typical form of a molecular force-field constituted by bonded (bond, angle, dihedral, and torsion) interactions and nonbonded (electrostatic and van der Waal’s) interaction energy terms. The force filed parameter set include: equilibrium bond length $\left(l_{0}\right)$, bond angle $\left(\theta_{0}\right)$, dihedral angle $\left(\omega_{0}\right)$, torsion angle ($\varphi_{0})$, and their respective force constants, $k_{l}$, $k_{\theta }$, $k_{\omega }$, and $k_{\varphi }$, as well as charge $(q_{i})\;$ on each atom i, dielectric constant $\left(\epsilon_{0}\right)$, strength of dispersion interactions $\left(\epsilon_{ij}\right)$, and contact distance $\left(\sigma_{ij}\right)$ between the atoms i and *j*. The interatomic distance is represented by $r_{ij}.$

The classical atomistic MD approach is to simulate each atom of the system using pairwise interatomic potentials to estimate the interaction strength of each atom with its remaining atoms in the system. The interaction energy of each atom in a system is modeled using a molecular force field, which comprises of both bonded (bond stretch, angle bend, dihedral rotation, and torsional) and non-bonded (electrostatic and van der Waal’s) interaction terms (Figure 4). There are many force fields available in the literature for proteins, such as CHARMM [137], AMBER [149], GROMOS [150], and OPLS [151] among others. Each force field has a unique set of parameters that are optimized to reproduce experimental reference data. Therefore, selection of a force field is an important decision before starting an MD simulation for any system.

The all-atom simulations are restricted to small time-steps (1–4 fs) to capture the intra- and intermolecular motion of atoms over time. Atomistic simulations result in detailed structural, thermodynamic, and kinetic data that can be compared to experimental data. A typical biomolecular system comprises of millions of atoms, embedded in ionic media to mimic the physiological conditions, simulating biological processes over micro- or millisecond timescales. Although atomic- level molecular modeling provides specific interatomic interactions, its practical application is limited by computational resources.

Setting up membrane protein systems for molecular simulations is challenging when it comes to embedding a protein in multicomponent lipid membranes. To streamline the process, the recent development of publicly hosted interactive web-based servers (such as charmm-gui) [152] is enabling the generation of input files required for membrane protein simulations. Among the existing simulation packages are popular MD engines such as GROMACS [153,154], NAMD [155], AMBER [156,157], CHARMM [152,158,159], and visualization tools such as VMD [160], Pymol [161], and YASARA (Figure 3) [162,163,164].

The use of CG simulations has increased in the last 10 years with the development of several force fields for a variety of biomolecules. Popular CG force fields include MARTINI [165,166,167,168,169], SIRAH [170,171], PRIME [172,173], and PACE [174,175]. Among these, MARTINI is the most popular for membrane protein simulations [165,166,176]. The MARTINI force field maps ~4 nonhydrogenic atoms into one bead; for example, a glycine amino acid residue is mapped as one bead [165,166,168]. By mapping a small number of atoms into a bead, CG resolution reduces the number of particles in a simulation and allows the system to be sampled less frequently with a larger time-step (20–40 fs). This approach enables simulation timescales that are 2 to 3 orders of magnitude larger than atomistic simulations. Thus, the advantages of CG simulations have spurred interest in simulations of larger sizes (hundreds of nanometers) and longer timescales (tens of microseconds) [177,178,179,180]. Others and we have rigorously demonstrated the ability of MD simulations to capture residue-level precision in membrane protein interactions [91,94,97,108,110,115].

### 4.3. Protein–Protein Interactions

#### 4.3.1. Self-Assembly Simulations

Molecular self-assembly simulation is a powerful technique that mimics interactions among molecules over time [94,98,145,181]. These simulations capture the spontaneous organization of molecules dictated by intra- and intermolecular nonbonded interactions (van der Waals forces, electrostatics, hydrogen bonds, hydrophobic, and hydrophilic interactions) under near-equilibrium conditions. Self-assembly simulations are routinely used to study complex membrane protein–protein interactions. These simulations, however, have several limitations including high computational cost. Membrane protein assembly is particularly computationally expensive in part because protein diffusion requires much longer equilibration time than the relaxation of membrane lipids. Moreover, the simulations need to be performed over a sufficiently long time to obtain reliable data, although ensuring convergence of such simulations is a challenge. There is no guarantee that a 10–100 µs timescale is long enough for proteins to assemble [182,183]. Employing multiscale simulations mitigates the challenges of long timescale simulation.

#### 4.3.2. Molecular Docking

Molecular docking, another popular method of studying protein associations, is a highly effective method to examine and rank interactions based on rigorous algorithms that evaluate empirical energetics data. Docking approaches are simple geometry-based methods where tens of thousands of protein–protein interfaces are generated via geometric rigid body motions followed by a scoring scheme where interfaces are ranked based on the energetics [184,185]. The energy function that is used in the scoring algorithm is often empirically modeled or derived from a knowledge-based method. Docking approaches tend to be fast because generating geometries of proteins is a simple task. Depending upon the type of protein–protein interaction, docking methods can give realistic results. Popular docking methods include Cluspro [186,187], Rosetta [188], HADDOCK [189,190], and ZDOCK [191] among others [192,193,194,195]. Cluspro has robust reproducibility, and it is the current state of the art in protein–protein docking. Rosetta is a more advanced software suite of protein modeling and analysis tools. The Rosetta docking protocol has been optimized for membrane proteins, and one can define membrane domain boundaries to guide the docking. However, despite the ease and speed of docking methods, static docking is not optimal for macromolecular assemblies that involve multiple proteins [196]. Moreover, for membrane proteins, the presence of the lipid environment significantly influences proteins’ association patterns, but none of the currently available docking methods offers a realistic lipid environment for protein docking [197,198,199,200,201,202].

#### 4.3.3. Protein Association Energy Landscape

An alternate strategy to study membrane protein association is to employ ensemble-based sampling methods. These methods use hundreds to a few thousand starting structures that each run independently to yield an unbiased sampling of the protein–protein association landscape. One such ensemble-based method, docking assay for transmembrane (DAFT) components [203] uses the population density of the resulting protein association conformations as a proxy for stability. Other ensemble-based methods use amino acid contact-based stability as a metric [204,205]; however, none of the available methods provides the association energies needed to determine the relative stability of protein–protein conformations obtained in the ensemble.

In our work, we used a combined stochastic sampling and MD approach to sample pairwise protein–protein interactions [206]. Taking advantage of in-plane diffusion of membrane proteins relative to each other in the lipid bilayer, we defined a two-dimensional rotational space of the interacting proteins. We adopted a stochastic sampling technique to generate thousands of seed geometries that uniformly sample the rotational space around a pair of interacting proteins. These seed geometries were then propagated independently to equilibrium using standard MD. The interaction energy associated with each conformation visited in the dynamics was computed to generate the protein association energy landscape (PANEL) [206]. By placing the proteins within the interacting distance of the seed geometries, we eliminated the need for protein diffusion, thereby reducing the simulation times for each system and boosting the PANEL performance by two orders of magnitude over classic self-assembly simulations. The PANEL method also provides an absolute measure of interaction energy for any pair of interacting proteins in dimeric or even in trimeric association [206]. We used the PANEL method to report the landscape of claudin-5 dimers (Figure 3), corroborating our previous self-assembly simulation results [115]. Furthermore, the robustness, computational affordability, and user-friendly implementation of the PANEL method makes it attractive for application to any membrane protein.

### 4.4. Pore Characterization and Paracellular Transport

The molecular architecture of the TJ pores and barriers are the determinants of the charge and size-selective transport of ions and molecules. Analysis of the pore’s structural features is important to understand TJ selectivity. The structural characteristics of TJ pores (such as length, diameter, volume, and cross-sectional area) provide valuable insight into the size selectivity of the pore. Additionally, identification of pore-lining residues in the channel walls and the bottleneck regions of the pore offer significant clues about their charge selectivity. Several software tools, such as CAVER [207], MOLE [208], HOLE [209], MOLAXIS [210,211], and POREWALKER [212], (each based on slightly different algorithms) provide structural analysis of protein pores (Figure 3).

Paracellular transport involves calculation of the free energy profiles of the translocation of a substrate through the pore. This calculated energy directly correlates to the permeability of the substrate across the pore [213,214]. The free energy profile can be obtained from the advanced sampling techniques of umbrella sampling [215] or metadynamics [216,217,218]. In the umbrella sampling technique, where multiple frames or conformations of a solute are generated along the length of the pore, an external force-constant is applied on the solute to restrain its position. Based on the amount of force applied, the free energy profile along the transport path can be evaluated [215,219]. An alternative method to determine the free energy profile is the recently introduced metadynamics approach where the system is biased gradually such that the ion translocation is made feasible within the affordable timescale of MD simulation. Metadynamics is available in GROMACS through the PLUMED plugin [220].

A simplistic equilibrium MD simulation can also be set up with different ion concentrations to observe the number of ion translocation events that occur during the simulation. This method may provide insights about preferred ion transport under unbiased, equilibrium conditions; however, the limitation of the method is the timesale in which an ion transport event will occur may not be within the simulaion time frame. To accelerate the ion transport event, computational electrophysiology has emerged as a method that involves biasing the molecular system by creating localized charge imbalances in system, which induces ion transport [221,222]. The small charge imbalances provide biologically realistic electrochemical gradients across the pore and facilitate ion transport.

## 5. Computational Nanoscopy of Claudin-5 in BBB Tight Junction Architecture

Claudin proteins embedded in the lipid bilayer undergo cis assembly via their transmembrane helical domains, or by the extracellular loops, or by both [94,115]. In general, protein–protein cis interactions are mediated by a multicomponent mixture of saturated and unsaturated lipids with variety of charged head groups and lipid tail lengths [91,106,201,202,223]. The helical bundle of membrane proteins has a rigid tertiary structure that provides stability to the protein to reside in the membrane environment. This rigidity in turn affects the local lipid environment and manifests as a hydrophobic mismatch between the protein and the lipids [198,224,225]. Hydrophobic mismatch is energetically unfavorable and serves as a driving force for the embedded protein to find interactions that would minimize its hydrophobic mismatch with the lipids and undergo cis assembly [103,226,227,228,229,230].

There are multiple aspects of claudin cis and trans assembly that influence the TJ architecture. The cis-assembled claudins can provide the template for the trans interactions [97,231,232]; therefore, the study of cis interfaces is vital to understand the TJ architecture. Besides, claudins are reported to be post-translationally modified (PTM) with phosphorylation and palmitoylation. Of these PTMs, studies suggest that palmitoylation alters recruitment of claudin proteins in the TJs and TJ permeability [91,233,234]. Since most aspartate–histidine–histidine–cysteine (DHHC) enzymes that assist in palmitoylation [235] are thought to be localized to the endoplasmic reticulum (ER) [198,236], it is likely that claudin cis interactions that are influenced by palmitoylation are localized in the ER. However, DHHC enzymes have been reported in other organelles and in the plasma membrane [237], which makes both the location of claudin-5 palmitoylation in the secretory pathway and the cis dimerization ambiguous. It is not surprising that the complexity of the BBB TJ architecture has been a deterrent in unraveling the mystery of the size and charge selectivity of the TJ. A list of basic questions that have confounded experts in the TJ research field include:

(1) How do claudin proteins form contiguous strands? Do the claudin proteins have preferred cis conformations?
(2) Where along the secretory pathway do claudin-5 proteins undergo cis assembly?
(3) How do posttranslational modifications of claudin-5 influence the cis interactions?
(4) How are TJ pores and barriers formed?
(5) How do BBB TJs get their size and change selectivity?

**Figure 5:**
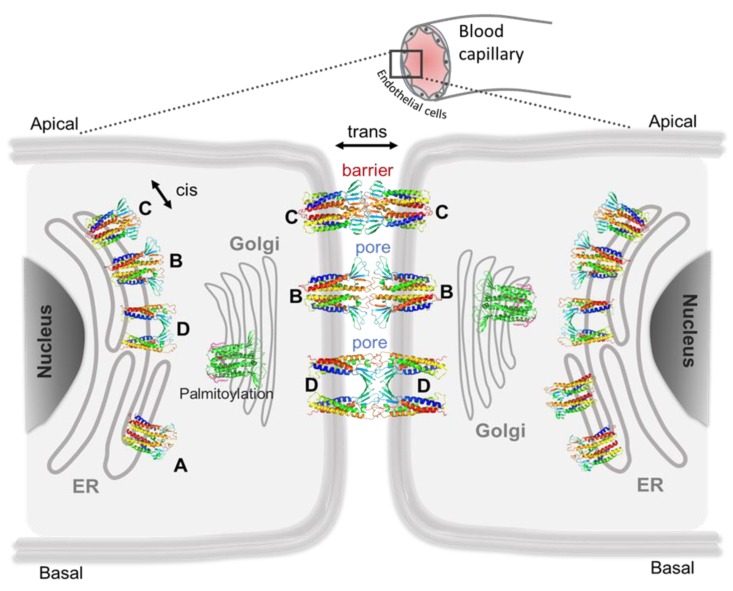
A schematic view of claudin-5′s cis and trans assembly along the secretory pathway. The cis dimerization of claudin-5 proteins in endoplasmic membrane (ER) and Golgi complex membrane can result in multiple conformations that are categorized as dimers A, B, C, and D [94,97,115]; posttranslational modification can influence the relative stability of these dimers [91]; the trans interaction of C dimer pair results in a paracellular barrier, whereas trans interaction of B–B or D–D dimer pairs result in paracellular pore.

To provide a new perspective to these long-standing challenges, we developed tools for in silico investigation of the TJ claudin-5 proteins. In a series of publications over the past few years [91,94,97,115,206], we have reported on the various aspects of claudin-5 BBB TJ architecture. Here, we reviewed the outcomes of these five lines of inquiry (Figure 5).

### 5.1. Claudin-5 Forms Symmetric and Asymmetric Dimer Conformations that Form Contiguous Strands

The cis interactions among claudins, determined by computational methods, corroborate results of biochemical assays [115]. The claudin-5 cis interactions were studied using both the self-assembly and the PANEL approaches [115,206].

**Figure 6:**
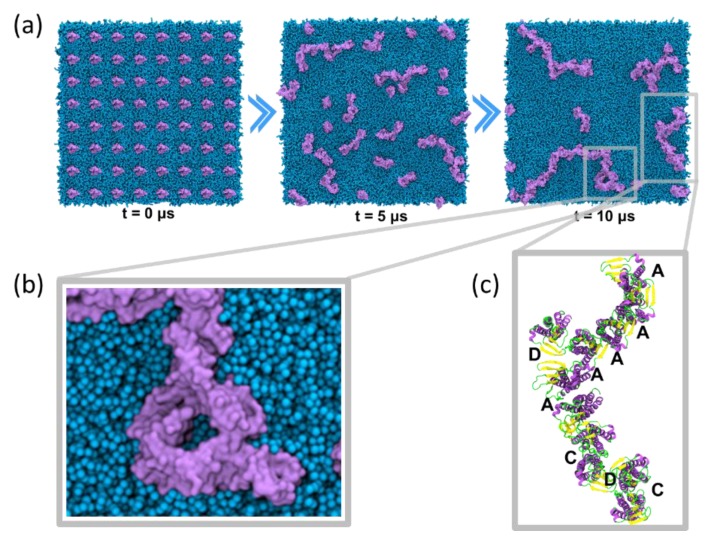
Claudin-5 strand formation in coarse-grain molecular dynamics (CG MD) self-assembly simulations. (**a**) Snapshots (top view) of claudin-5 (purple beads) assembly in lipid (cyan beads) membrane at *t* = 0, 5, and 10 µs, (**b**) close up view of a self-assembled claudin-5 pentamer, and (**c**) reversed-mapped strand showing dimer A, C, and D conformations.

The self-assembly simulations, performed in CG resolution, suggest that claudin-5 monomers embedded in a simple mixture of saturated and unsaturated lipids readily form dimers (within 400–500 ns) [115]. These dimers subsequently (in 5–10 µs) form contiguous strands and high-order assemblies (Figure 6a). For example, in a system composed of 64 claudin-5 monomers, a contiguous strand of 36 monomers was observed with a ring-like pentamer (Figure 6b), which match the size description of ~10 nm particles observed under freeze fracture [238,239,240]. The claudin strands present at the end of the 10 µs simulation were extracted and reverse mapped to atomistic resolution for conformational and structural analysis (Figure 6c). A pair-wise conformational analysis of the self-assembled claudins revealed four, most prevalent conformations (labeled dimer A−D) that concurrently exist to form the strands. Several repeat simulations have been performed that all show formation of dimers A−D although in varying populations. Since it is practically impossible to achieve convergence in self-assembly simulations due to finite timescales, we were unable to provide converged relative populations of each of the four dimers. However, these simulations indicate that a dimer is the smallest stable unit of the self-assembled strand and that the cis assembly can occur in the absence of trans interacting partners from the adjacent cell. In an experimental study, claudin-2 cis dimerization was also observed independent of cell-surface expression and intercellular cross-linking [241].

**Figure 7:**
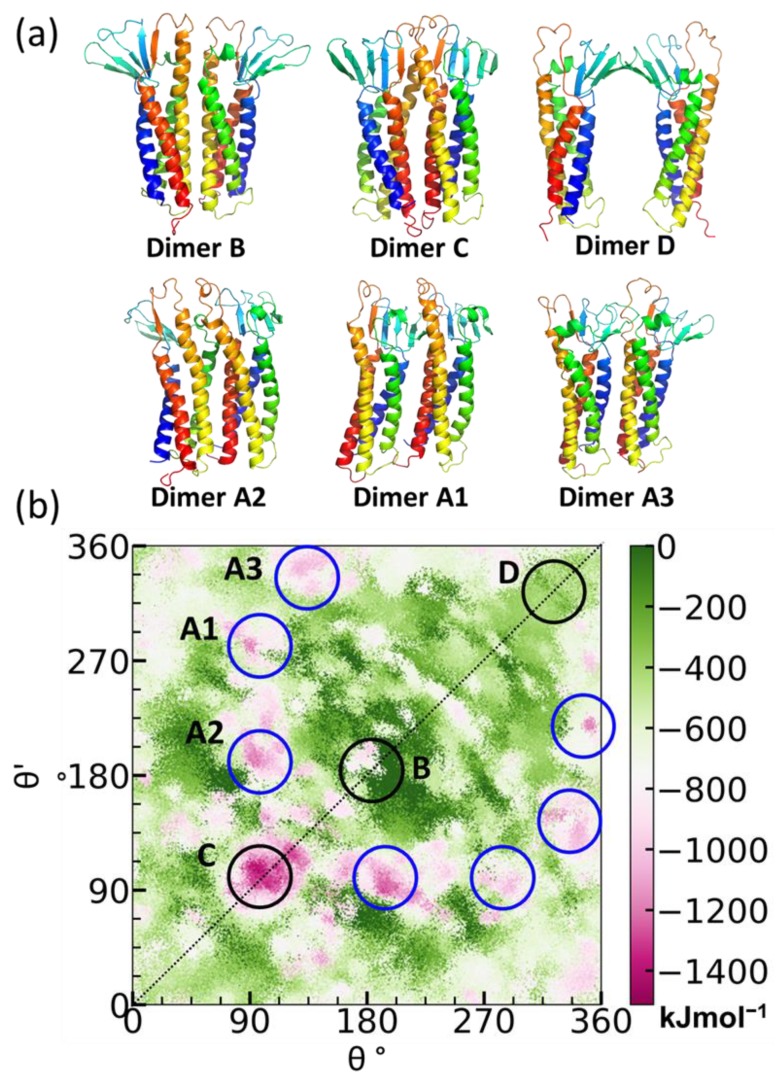
Claudin-5 cis dimerization. (**a**) Key cis dimers formed in claudin-5, reverse mapped to atomistic resolutions, and (**b**) PANEL plot showing location of low energy dimers A1, A2, and A3 (blue circles); and dimers B, C, and D (black circles).

To delve deeper into the stability of the claudin-5 dimers, we developed the PANEL approach [206]. The PANEL plot for claudin-5 provided a comprehensive sampling of dimer conformations and a clear visualization of the entire energy landscape as a function of the orientation angles (Figure 7). The claudin-5 dimer interaction energies range from 0 (least stable) to approximately −1500 kJ mol^−1^ (most stable) [206]. The PANEL contour plot shows multiple low energy conformations. Conformations along the diagonal (Figure 7b) represent symmetric dimers, while those off the diagonal are asymmetric dimers. It was reassuring that the key symmetric Dimers B, C, and D, also observed in self-assembly and in experiments, are shown in the minimum energy PANEL plot. Dimer B is a TM3:TM3-mediated dimer, while Dimer C is mediated by TM4:TM4, and Dimer D, the symmetric ECL1-mediated interaction, also proposed by Suzuki et al. [242]. There are also several asymmetric dimers (Dimer A), which are marked as Dimers A1, A2, and A3 in the PANEL plot. Dimer A1 corresponds to the linear arrangement observed in the claudin-15 crystal structure [242]. The relative orientation of the claudins in each of the Dimers A to D (Figure 7b) is described in our earlier work [115,206]. Although the presence of claudin dimers and higher order complexes has been reported in several experimental studies [73,240,241,243,244], isolating intact dimers or higher order complexes still remains a challenge. Use of computational approach provides a direct look into the structural details of these claudin interfaces.

### 5.2. Primary Cis Interactions Occur Early in the Secretory Pathway

Like other TJ proteins, claudins are secreted in the endoplasmic reticulum (ER) and reach the plasma membrane via the Golgi complex [231] Along this secretory pathway, the membrane composition changes drastically [245,246,247] To mimic the complexity of cell organelles encountered by a typical membrane protein during its secretory pathway, we performed claudin-5 self-assembly in three model membrane compositions: ER, cholesterol-enriched endoplasmic reticulum (ERc), and plasma membrane (PM) [91]. The ER membrane model constituted a binary mixture of saturated and unsaturated lipids in 1:1 ratio; the ERc was a 2:2:1 ternary mixture of saturated lipid, unsaturated lipid, and cholesterol; and the PM model comprised ten lipid components including gangliosides, sphingomyelin, and cholesterol [91].

**Figure 8:**
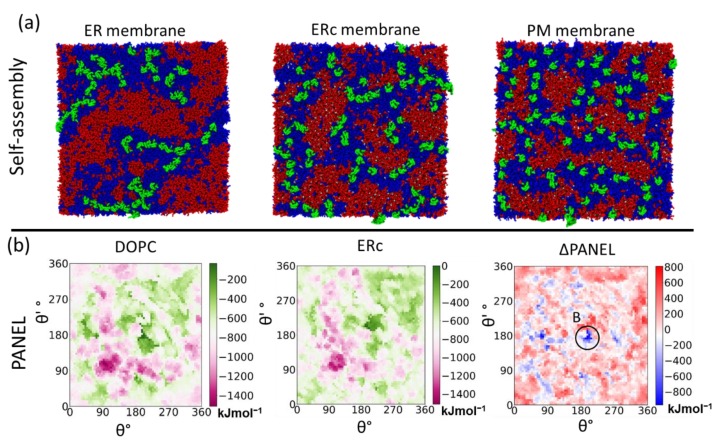
Effects of lipid complexity on claudin-5 dimerization. (**a**) Snapshots (top view) of self-assembled claudins (green) embedded in ER, cholesterol-enriched endoplasmic reticulum (ERc), and plasma membrane (PM) membranes with saturated lipids (red), unsaturated (blue), and cholesterol (white). (**b**) PANEL plots generated for claudin-5 dimers embedded in symmetric DOPC bilayer and ERc membrane. The ΔPANEL plot shows stabilization (blue) and destabilization (red) of the dimer geometries due to change in lipid environment DOPC to ERc. The stabilized dimer B location is marked (circle, black) on the ΔPANEL plot.

Our findings indicate that claudin-5 monomers are more likely to form dimers in simple membranes like the ER where saturated and unsaturated phospholipids are the principal components. As claudins move to the Golgi complex, trans-Golgi vesicles, and the plasma membrane, the higher cholesterol concentrations in these membranes limit claudin-5 assembly. We observed that an increase in membrane complexity was negatively correlated with the length of claudin-5 strands (Figure 8a) [91].

A comparison of PANEL plots for claudin-5 dimers in a simple unsaturated di-oleoyl-phosphatidylcholine (DOPC) lipid membrane and ERc membrane shows the impact of the lipid composition on dimer stability (Figure 8a). The effect is quantified by taking the difference in interaction energies (ΔPANEL) between corresponding geometries (*θ*, *θ*’) on the two plots. A vast majority of the dimer interactions were destabilized in ERc membrane, although a few conformations did show more favorable interactions. In particular, Dimer B conformation (mediated via TM3:TM3 interaction) is stabilized. Overall, our results suggest that dimerization is more favorable in conditions existing in the early stages of the secretory pathway.

### 5.3. Posttranslational Modifications Alter Relative Stability of cis Dimers

Several studies have indicated the significance of palmitoylation in the recruitment of specific claudins to TJ and the consequent effect on the TJ permeability [233,234]. Since palmitoylation and depalmitoylation are highly labile reactions, the evaluation of these modifications on claudin assembly is experimentally challenging [237]. However, in silico methods provide the necessary spatial and temporal resolutions required to study these posttranslational modifications.

**Figure 9:**
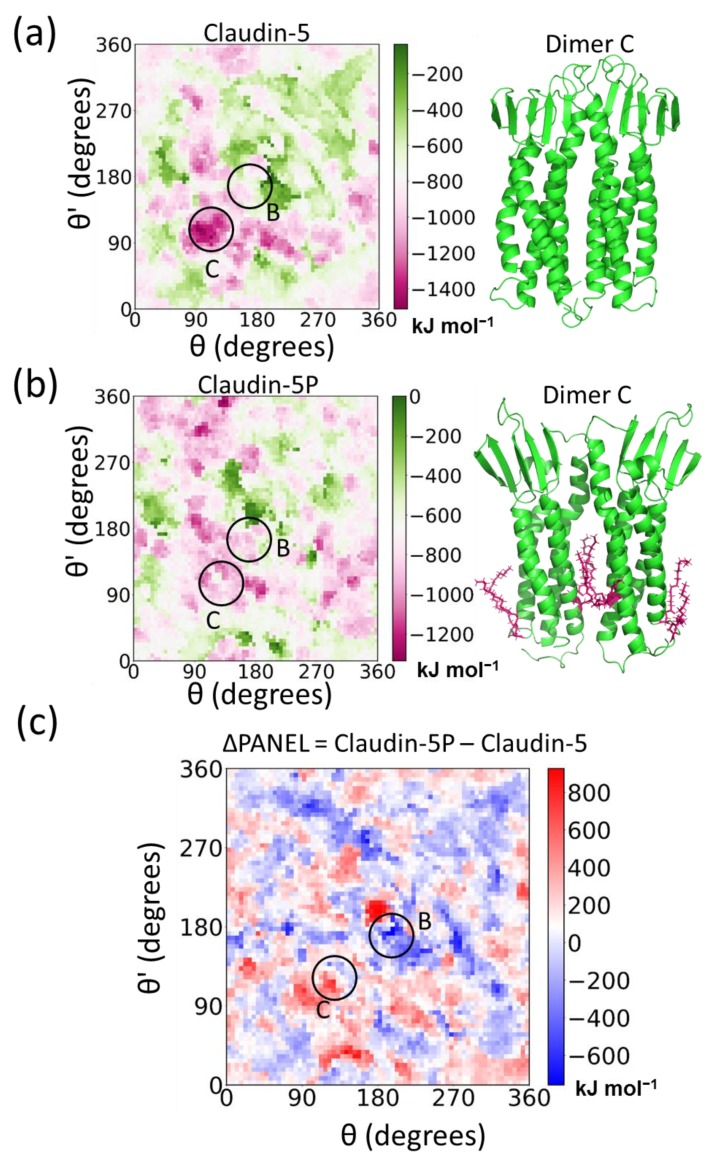
Effects of palmitoylation on claudin-5 cis dimerization. (**a**) PANEL plot Figure claudin. and dimer C conformation (green ribbon), (**b**) PANEL plot for claudin-5P and dimer C conformation (green ribbon) with palmitoylation (pink sticks), and (**c**) ΔPANEL showing the difference in stability upon palmitoylation. The location of dimers B and C are marked by circles (black) on all plots.

The presence of palmitoyl chains at the protein–lipid interface introduces variations in interfacial properties, effecting both the extent of dimerization as well as the distribution of specific dimer interfaces. Our results show that palmitoylation increases cholesterol and saturated lipid accumulation around the claudins and enhances cis interactions to the Dimer B interface while disrupting Dimer C largely due to steric factors (Figure 9a,b) [91]. The impact of palmitoylation on claudin-5 cis dimers is evident from the ΔPANEL landscape plotted as the difference between palmitoylated claudin (claudin-5P) and non-palmitoylated claudin-5 PANEL plots (Figure 9c). The ΔPANEL plot shows the stabilization of Dimer B and the destabilization of Dimer C regions. The change in dimer stability upon palmitoylation indicates that posttranslational modification can be a potential strategy to control paracellular permeability [91,97,233,234,237,248,249].

### 5.4. Cis Dimers Act as Precursors to Form Trans Interfaces

Trans interactions are formed when claudins on adjacent cells interact head-on to form the macromolecular TJ assembly. We adopted both CG- and atomistic-level simulations to decipher the nature of trans interfaces. Numerous challenges were posed both on conceptual and computational resource fronts while designing the trans assembly simulations.

**Figure 10:**
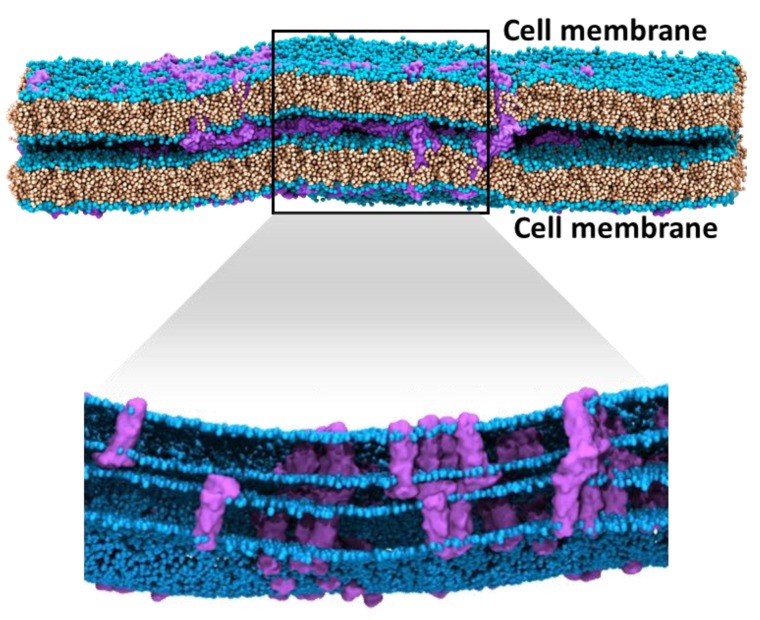
Claudin-5 assembly in two adjacent membranes. A 20 µs snapshot of claudin-5 (purple) trans and cis assembly in a coarse gained MD simulation. The zoomed-in view shows the interacting claudins embedded in the membrane (lipid head groups (blue); lipid tails (light brown). Water and ions in the system are not shown for clarity.

To mimic paracellular space, our initial studies of trans interactions used two membrane patches with multiple (64) equidistant claudin-5 proteins [97]. They were placed head-on, such that the ECL loops from either membrane could interact within the gap between the two membranes (Figure 10). Sampling 20 µs of trans self-assembly took about 12 days on a supercomputer using 20 high performance computational nodes. Despite the intensive computation, the simulations represented only a small membrane patch that contained 128 claudin monomers across the two membranes. In contrast to a single membrane simulation, trans assembly simulations were unable to capture a few cis dimeric interactions that were prevalent in the single membrane self-assembly simulations. The simulations were intentionally designed so that a diffusing claudin-5 monomer would encounter a trans interacting partner before encountering a cis interacting partner; this ensured generation of more trans interaction variants. The absence of cis dimers rendered this a monomer-driven, trans interaction assembly [97]. Multiple pore-like assemblies were observed in these simulations showing ECL contacts stabilized by hydrogen-bonds and salt bridges. These trans pairs also strongly resembled pore models suggested in previous studies [72,73,74,250]. However, this model represented a very low fraction of the actual trans interactions, which span the entire perimeter of a cell and involve tens of millions of protein monomers.

From the insights obtained from the cis and trans self-assembly simulations a dimer-guided trans interaction study was carried out using molecular docking approaches. This method yielded highly promising pore-forming trans interfaces. Moreover, the formation of cis dimers before trans interactions has been suggested in several findings [231,241,251,252]. Based on our work, we suggest that the cis dimers act as precursors for forming trans interfaces [91,97,115]. The fact that cis assembly simulation also self-assembles into a dimer in shorter timescales, lends support to that cis interactions may act as the template to guide the trans assembly.

The findings from these simulations were able to fill significant gaps in our understanding of the structure–function relationships between claudin monomers. Although several questions still need to be answered, and further investigations need to be performed to achieve a comprehensive understanding of the TJ architecture, our current understanding has been made possible by the an array of techniques in the computational toolkit.

### 5.5. Sub-Nanometer Wide Claudin-5 TJ Pores, Lined by Charged Residue Determine Pore Selectivity

**Figure 11:**
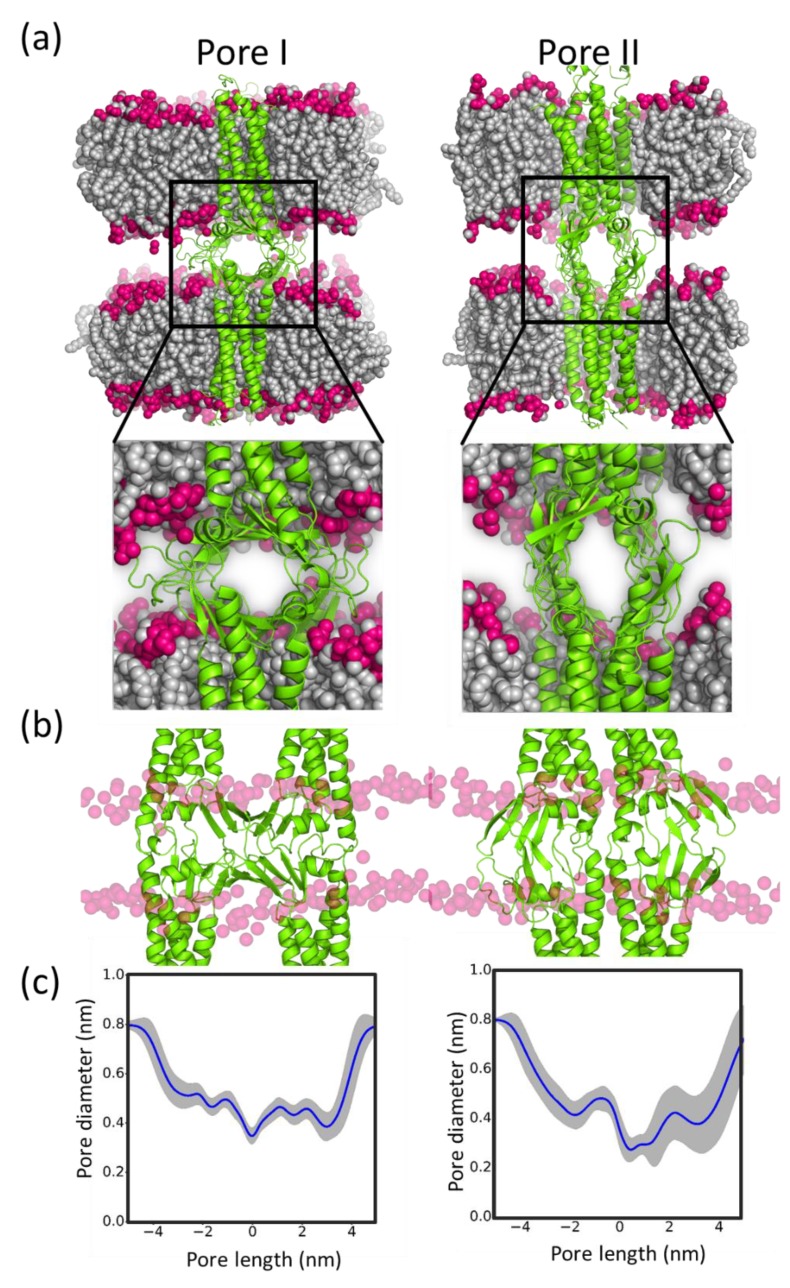
Model for claudin-5 pore I (left) and pore II (right). (**a**) Front-view, (**b**) side-view, and (**c**) pore diameter along the length of the pore.

For claudin-5, two pore models, mediated by different cis dimers, have been reported based on trans interaction simulations and mutation analyses of cis dimers [97]. The pore I model is a face-to-face stacking of the dimer D interface. This pore resembles the proposed claudin-15 pore [242]. The pore II model was based on the Dimer B interactions observed in claudin-5. These models were generated using docking methods in combination with symmetric refinement to arrive at the pore structures based on Dimers D and B (Figure 11). Fundamental pore features, such as diameter, length of the pore, pore bottleneck diameter, and the pore lining residues, were characterized.

Pore characteristics provide a general idea of the nature of the pore. However, evaluating the energetics of molecular transport along these pores is essential to further enhance our understanding of paracellular selectivity of the BBB’s TJs. Using advanced sampling methods, translocation of water and glucose was reported across claudin-5 pore model formed by Dimer B. These simulations showed that the claudin-5 Dimer B pore allows water to translocate without energy barriers but had a higher energy barrier for glucose translocation compared with the GLUT1 glucose transporter [97,253,254]. Further extension of this work revealed that claudin-2 is selective to sodium ions with multiple observations of ion translocation under equilibrium conditions. Similar approaches are gaining attention within the field (e.g., claudin-15 pores probed using MD simulations) [255,256,257]. The more comprehensive undertaking of looking at ion transport in pores formed by different claudins is still in its infancy, but several computational groups are developing the essential methods needed to pursue this line of inquiry. In the meantime, the proposed pore structures present opportunities for further refinement to obtain better insights about their structural as well as functional aspects.

## 6. Additional Factors that Influence Tight Junctions

Computational methods can efficiently study cis and trans interactions of claudins at the molecular level. Claudins and other TJ proteins exist in a chemical environment that varies in type and amount of proteins and lipids, hydrophobicity, protein lipidation states, and other aspects [63,200,225,231,246,247]. These physiological factors contribute to the final TJ architecture. Moreover, TJs are known to be dynamic entities that undergo constant structural change [258,259]. Thus, to understand paracellular transport in its entirety, other factors need to be considered. A few key aspects important to TJ formation are discussed below.

### 6.1. Higher Order Claudin Aggregates

Several studies have reported the presence of claudin aggregates (in addition to strands) [61,118], suggesting that higher-order aggregates such as trimers, tetramers, or hexamers may be formed. *Clostridium perfringens* enterotoxin, which causes food poisoning, has been reported to form higher-order aggregates upon interacting with claudins. We proposed that the trimeric form of the enterotoxin could potentially bind to the trimeric form of claudins in the intestinal epithelium [94,117]. In another recent report of claudin-11 TJs, the carbon replica method revealed an interesting view of consistent double-stranded structures [260]. These reports emphasize that in order to fully understand the architecture of the TJ strands, it is important to consider the higher-order interfaces in addition to dimers.

### 6.2. Involvement of Other TJ Proteins: Key to TJ Structural Stability

In addition to claudins, proteins from approximately 80 other families are known to be involved in the TJ protein complex assembly including occludins and JAMs [53,54,60]. Occludins and JAMs contribute to the structural stability of TJs. Although these proteins are not known to affect directly TJs’ selective permeability or barrier function, their role in maintaining the structural integrity of the TJ strongly suggests they form interfaces with claudins in order to support the strand. This may lead to masking of some claudins’ interfacing surfaces while binding to others, thereby redistributing the relative populations of dimer interfaces. The study of occludins and JAMs is crucial to provide a complete perspective of TJs due to their significant presence within the TJ assembly.

### 6.3. Cytoskeletal Involvement

Claudins and other TJ membrane proteins are dynamically regulated by scaffolding proteins (zonula occludens-1/2/3) and actin cytoskeleton [53,231]. Although studying claudins individually provides insights about their interface forming behavior, thereby helping us to predict the architecture and nature of the corresponding TJ strands, continuous regulation by the cytoskeleton may induce several factors such as localized redistribution of claudin populations, or regulation of biophysical properties such as protein tilting, membrane anchoring, and so on. Since the cytoskeletal regulation tends to be highly dynamic and specific to the needs of the cell, deciphering their roles remains one of the greatest challenges.

## 7. Future Work

Future work on the BBB research will be directed towards the development of strategies to enable and enhance drug delivery to the brain. A fundamental understanding of the molecular construct that guards the blood–brain interface has been obtained through computational tools, which have enabled the design of in silico models to explore and the understanding of the architecture of this interface. One future research goal is to identify small molecules that can act as TJ modulators for use in screening claudin-5 trans interactions, reducing specificity, increasing pore size, and enhancing pore permeability in a controlled and reversible manner. Having the molecular-level pore structure is foundational to this endeavor. We envisioned that in vitro studies would be designed in the future to validate the predictions of the computational models. Other future work includes pursuing an integrated computational and in vitro/in vivo study of multiple drug pathways for neurotherapeutics, evaluating molecular drug interactions, obtaining the dynamics of transport of potential drug molecules with the vision of quantifying drug permeability through the BBB.

## Abbreviations

BBB	Blood–brain barrier
CG	Coarse grained
Claudin-5P	Palmitoylated claudin
DAFT	Docking assay for transmembrane
DHHC	Aspartate-Histidine-Histidine-Cysteine
DOPC	1,2-dioleoyl-sn-glycero-3-phosphocholine
ECL	Extracellular loop
ER	Endoplasmic Reticulum
ERc	Cholesterol enriched Endoplasmic Reticulum
ICL	Intracellular loop
JAMS	Junctional adhesion molecules
MD	Molecular dynamics
PANEL	Protein Association Energy Landscape
TEER	Transendothelial/epithelial electrical resistance
TJ	Tight junction
TM	Transmembrane loop
ZO	Zonula Occuldens
